# Restoration and Evolution of the Paleogene (E_1_f_2_) Shale Sedimentary Environment in the Subei Basin, China

**DOI:** 10.1021/acsomega.3c06603

**Published:** 2023-11-30

**Authors:** Qiang Fu, Zongquan Hu, Dongjun Feng, Jianling Huang, Lele Xing, Zhiwei Zhu, Wen Teng

**Affiliations:** † State Key Laboratory of Shale Oil and Gas Enrichment Mechanisms and Effective Development, Beijing 100083, China; ‡ Sinopec Key Laboratory of Shale Oil/Gas Exploration and Production Technology, Beijing 100083, China; § State Key Laboratory of Marine Geology, Tongji University, Shanghai 200092, China

## Abstract

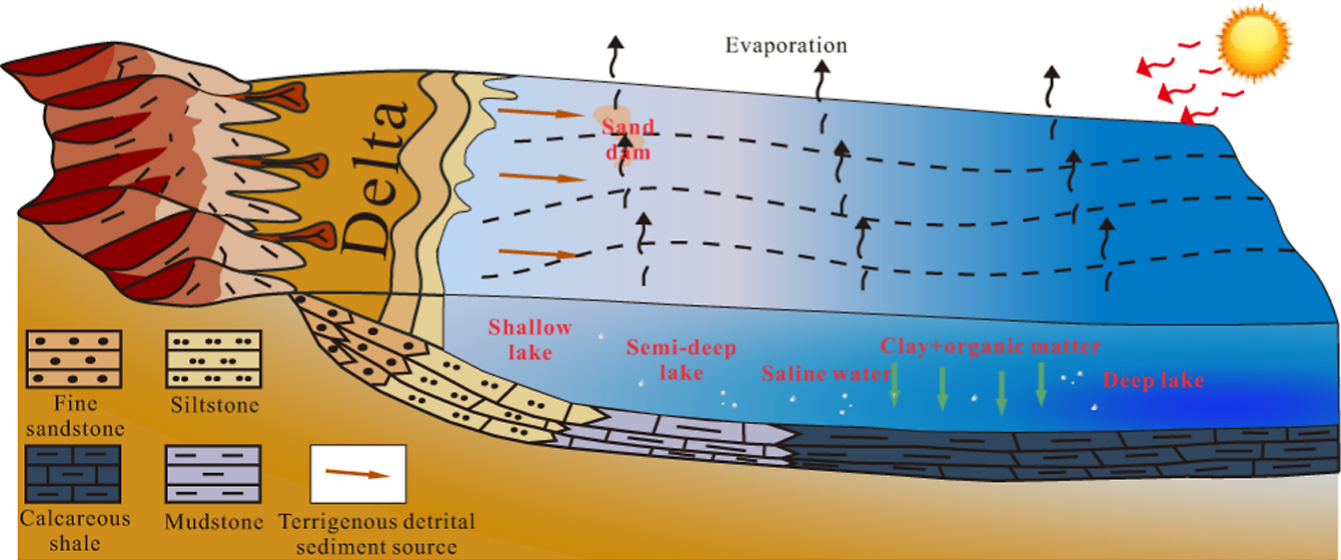

Restoring the sedimentary environment of paleolakes is of great significance to the formation of laminated calcareous shale deposited in paleolakes and the prediction of shale oil reservoir distribution. This article focuses on the second section shale of the Paleogene in the Funing Formation in the Gaoyou Sag, Subei Basin, China, and uses X-ray fluorescence diffraction technology and core lithology analysis methods to obtain the content datum of major and trace elements such as Sr, Cu, Ba, Ga, V, and Ni in shale at different depths. Based on the empirical values of Sr/Cu, Sr/Ba, V/(V + Ni), and total organic carbon, paleoenvironmental evolution of the continental shale was determined and studied, including the changes in paleoclimate temperature, paleosalinity, paleowater depth, and strong or weak redox intensity. The research results indicate that the sedimentary environment of the paleolake in the Paleogene Funing Formation, second section, in the Gaoyou Sag is mainly characterized by a dry and hot climate; the salinity of paleolake water is that of stable brackish water, and the entire sedimentary period of the Funing Formation, second section, is dominated by a reduction environment, which is conducive to the preservation of sedimentary organic matter. The frequent changes in the depth of sedimentary water and the alternating dry and hot climate are the main reasons for the development of laminated calcareous shale in the second section of the Paleogene Funing Formation of the Gaoyou Sag and have also contributed to the abundant commercial resources of laminated calcareous shale oil in the second section of the Funing Formation.

## 1 Introduction

The use of laboratory analytical instruments for analyzing and testing rock samples has always been an important means of obtaining the composition and abundance of major and trace elements in rocks.^1,2^ However, due to limitations such as high prices and testing cycles, it is difficult to analyze the composition and abundance of major and trace elements in rocks using measured data from a large number of samples in practical work. After more than 50 years of development, X-ray fluorescence (XRF) spectroscopy analysis has the characteristics of high testing accuracy, high vertical resolution (1 cm), small errors, and nondestructive testing of rock samples compared to traditional methods for major and trace element testing. Therefore, it has been widely applied in research such as core and debris logging analysis, paleoenvironment and paleoclimate restoration, high-resolution sequence division, diagenetic process research, and source analysis.^3^

Paleosalinity is a record of water salinity in ancient sediments and an important indicator of ancient sedimentary environments in geological history. High salinity is conducive to the preservation of organic matter. Based on the analysis of fossils, lithology, and trace elements in the second section of the Funing Formation, it is believed that there was a marine invasion event during the sedimentary period of the second section of the Funing Formation.^4^ There are many methods for paleosalinity discrimination, such as sedimentary phosphate method, isotope method, and trace element geochemical method for quantitative division of water salinity.^5−9^

The organic matter content [total organic carbon (TOC)] in shale is also closely related to the sedimentary environment and water depth. Generally, shallow lake areas have a large amount of terrigenous debris, and the organic matter is diluted, the lake water is turbulent, and the conditions of partial oxidation make it difficult to preserve organic matter, resulting in low total organic carbon.^10−12^ The semi deep lake is located below the base of storm waves, with quiet water, reduced terrestrial debris, and surface water depth suitable for the proliferation of plankton. After plankton die, they settle at the bottom of the lake in a reducing environment, making it easy to preserve organic matter, resulting in the highest TOC.^13^

The Subei Basin is a fault depression basin with a large scale, high lake level, and the characteristics of “underwater uplift”. The fault depression in the Eocene Oligocene has strong separation, which is significantly different from the early fault depression.^14^ The Eocene Oligocene fault depression has strong separation, which is significantly different from the earlier fault depression. The Funing Formation of the Paleogene in the Subei Basin exhibits a sand rock (E_1_f_1_)—shale(E_1_f_2_)—sand rock(E_1_f_3_)—shale(E_1_f_4_) rhythm from bottom to top. The second section of the Funing Formation(E_1_f_2_) is mainly composed of black laminated calcareous shale, gray thin siltstone, interbedded thin biological limestone, and a few oolitic limestones. The core description shows that during the sedimentary period of the second section of the Funing Formation in the Paleogene, the lake was rich in organisms, mainly including ostracods, gastropods, fish, spores, and pollen.^15^ There are currently seven wells producing commercial shale oil in the dark shale rich in organic matter in the second section of the Funing Formation, indicating that the black laminated calcareous shale in the second section of the Funing Formation has abundant shale oil resources.

The black laminated calcareous shale of the second section of the Funing Formation of the Paleogene in the Gaoyou Sag, Subei Basin, is a comprehensive result of three geological processes: sedimentation, tectonism, and diagenesis. Sedimentation is the original and decisive internal cause, while diagenesis and tectonism are late, affecting and reforming the original reservoir. The depth of the influence and transformation is constrained by the original sedimentation.^16^ Therefore, in order to study the formation and distribution characteristics of shale reservoirs in the second section of the Funing Formation of the Paleogene in the Subei Basin and to explore shale oil resources more accurately, it is necessary to understand and restore the paleolake environment and its evolutionary characteristics during the deposition of the shale.

## 2 Regional Geologic Background

### 2.1 Structural Background

**Figure 1:**
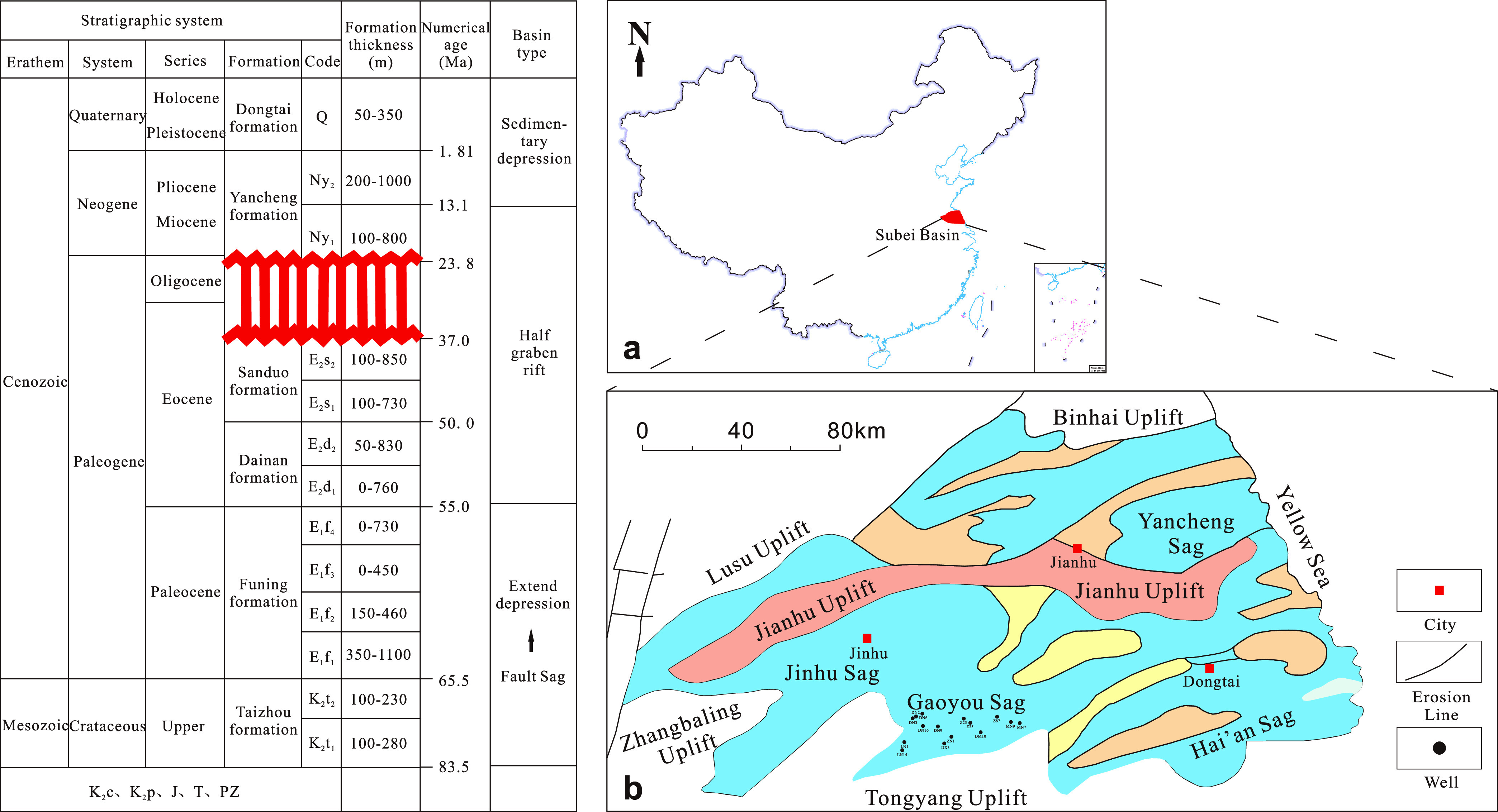
Characteristics and tectonic evolution of the Subei Basin. (a) Location of the Subei Basin. (b) Structural unit map of the Subei Basin.

Subei Basin is a continental oil-forming basin developed from the Yizheng movement in the Late Cretaceous on the basis of Mesozoic Paleozoic marine sedimentary basement. The basin is adjacent to the coastal uplift to the north, bounded by the Tongyang uplift to the south, adjacent to the Lusu uplift to the northwest, and reaching the Yellow Sea to the east, with an area of approximately 3.5 × 10^4^ km^2^ (Figure 1a). The interior of the basin can be divided into four secondary structural units that extend in an east–west direction, with Dongtai Depression, Jianhu Uplift, Yanfu Depression, and Binhai Uplift from the south to north. Each depression can be further divided into several secondary depressions and low uplifts. The basin has experienced four evolution stages, namely, the Late Cretaceous fault depression, Paleogene depression, Eocene fault depression, and Neogene and Quaternary thermal subsidence depression (Figure 1), where the upper Cretaceous, Paleogene, Neogene, and Quaternary sediments are widely developed.

Gaoyou Sag is located in the Dongtai Depression of the Subei Basin, with a length of about 100 km from east to west, a width of about 25–35 km from north to south, and an area of about 2670 km^2^. The depression is controlled by a large boundary fault in the south, with a dustpan-like fault depression structure characterized by a southern fault and a northern overlap. The northern part is connected to the Zheduo low protrusion by a gentle slope, while the western part is connected to the Jinhu Sag by the saddle between the Liubao low protrusion and the Lingtangqiao low protrusion. The eastern part is adjacent to the Qintong Sag. The sedimentary thickness of the Mesozoic and Cenozoic strata reaches 7000 m, making it the largest depression in the subsidence amplitude of the Subei Basin. The sedimentary rock series in the sag is developed, with a good oil-generation environment, many types of oil and gas reservoirs, and high enrichment degree. It is an oil-and-gas-bearing area with a high degree of exploration.

### 2.2 Sedimentary Stratigraphic Characteristics

The thickness of the second section of the Paleogene Funing Formation (E_1_f_2_) in the Gaoyou Sag is usually 200–300 m thick, with a maximum thickness of 370 m. It is a set of dark gray and grayish black thick shale interbedded with thin interbedding between marl and dolomite. Vertically, it can be divided into the following: the lower part is thick gray black shale interbedded with a thin layer of siltstone, thin layer of biological limestone, and thin layer of oolitic limestone. Since the resistivity logging curve has four high peaks, it is commonly known as the “four peaks” section; the middle part is the interbedded interval of thin grayish black shale and marl mixed with thin dolomite limestone. Because the resistivity logging curve presents seven high peaks, it is commonly known as the “seven peaks” section; the upper part is a relatively pure thick grayish black shale, commonly known as the “mudstone neck section”. The paleontological assemblages discovered in the strata are ostracods, including (*Homoeucypryscucerusa*), (*Pa railyocypryschngzhouensis*), and (*Sandona (Lineocypris) a cclina*), representing a high degree of fossil differentiation in the second segment of the Funing Formation; in addition, there are also combinations of *Sinocyprisreticulata* and *Moenocyprislepida* in the grid. Moreover, there are multiple phyla fossils such as polychaetes, foraminifera, fish, and calcareous nannofossils.

The strata of the second section of the Paleogene Funing Formation in the Subei Basin belong to semi saline open lacustrine deposits. Shale deposits with stable lithology are developed throughout the entire basin, and the electrical correlation marks are obvious for the shale. The “seven peaks” interval in the middle constitutes the second important stratigraphic division and correlation marker bed in the whole basin and has stable distribution throughout the basin. During the sedimentary period of the second section of the Funing Formation, the lake basin was relatively stable and the surrounding terrain had little elevation difference, basically maintaining a sedimentary pattern of high in the west and low in the east. The second section of the Funing Formation can be vertically divided into three sedimentary periods: the early stage, the beginning stage of marine invasion, wherein, due to relatively calm crustal movement, the land continues to decline. On the other hand, the weakening of erosion greatly reduces the amount of debris entering the lake, leading to the rapid expansion of the lake body to the entire basin. In the middle stage of the second section of the Funing Formation, the stage of transgression, the influence of transgression is further increased, the water body of the lake basin expands, the water surface rises, and the water depth increases. A set of thin grayish black shale with thin marl interbedding and uniform lithology is deposited, which is the product of semi deep-water environment. In the late sedimentary stage to regression stage of the second section of the Funing Formation, seawater gradually withdrew and continued to deposit a set of grayish black lacustrine shale (the “mudstone neck section”) on a flat terrain.

## 3 Samples and Methods

In order to analyze the sedimentary paleoenvironment and its evolution in the second section of the Funing Formation, this study used trace element analysis testing methods and data to restore the changes in the sedimentary paleoenvironment recorded by the changes in the constant and trace elements in shale. The shale mineral composition samples are from seven wells in the second section of the Paleogene Funing Formation in the Gaoyou Sag, and the data are from the experimental report analyzed by the laboratory of Sinopec Jiangsu Oilfield Exploration and Development Research Institute. The clay mineral and whole rock were tested using a D/Max-1200 type X-ray diffractometer, and the analysis is based on the oil and gas industry standard “Analytical Method of Clay Mineral and Common Non clay Mineral X-ray diffraction in sedimentary rock: SY/T5163-2010″.

### 3.1 Samples

All 30 experimental samples were obtained from the shale cores of the second section of the drilling Paleogene Funing Formation in the Gaoyou Sag, Subei Basin, two of which were provided by the State Key Laboratory of Marine Geology as test standard samples, and the remaining twenty-eight samples were used as test samples for the restoration of the paleoenvironment and paleoclimate. The experimental samples were tested using a laboratory XRF spectrometer, and the results are shown in Table 1.

**Table 1:** XRF Analysis Results of Samples from E1f2 in the Gaoyou Sag, Subei Basin

					element content (μg/g)						
formation	sublayer	**well No**	**depth(m)**	lithology	Sr	Ni	Ga	Sr/Cu	Sr/Ba	V/(V + Ni)	V/Cr
E_1_f_2_	four peaks	**DN2**	**1605.77**	silty shale	586	76	29	7.75	1.61	0.51	1.37
		**Z23**	**1744.31**	silty shale	1351	62	30	50.22	2.53	0.50	1.94
		**Z87**	**2385.23**	lamellated calcareous shale	1260	28	29	17.97	2.11	0.69	1.64
		**DN16**	**2386.1**	micritic dolomite	791	34	24	22.53	1.45	0.67	1.37
		**MN7**	**1744.24**	lamellated calcareous shale	1403	16	31	41.41	2.72	0.79	1.75
		**DN3**	**2658.5**	silty shale	2288	39	23	77.04	3.33	0.59	1.68
		**LN14**	**1391.05**	silty shale	1782	37	22	71.24	3.09	0.72	1.74
		**ZN1**	**1394.23**	silty shale	730	44	30	26.43	1.91	0.68	1.51
		**MN9**	**1567.6**	lamellated calcareous shale	196	34	23	1.95	0.82	0.66	1.17
		**DN9**	**1569.4**	lamellated calcareous shale	242	40	22	3.10	0.54	0.64	1.15
		**DN6**	**1572.8**	silty shale	521	34	21	6.67	1.01	0.64	1.05
		**DN6**	**1638.94**	lamellated calcareous shale	824	33	26	8.33	1.91	0.70	1.39
		**DN6**	**1639.94**	silty shale	711	31	28	9.10	1.37	0.73	1.43
		**DM10**	**1641.64**	silty shale	1680	20	23	17.17	3.48	0.79	1.86
		**average value**	1026	38	26	25.78	2.00	0.66	1.51		
	**seven peaks**	**DN2**	**1520.23**	lamellated calcareous shale	1575	42	29	21.42	3.04		
		**DN2**	**1522.34**	lamellated calcareous shale	1831	57	25	22.69	3.22	0.49	1.47
		**MN2**	**1542.99**	lamellated calcareous shale	845	81	27	11.34	2.15	0.48	1.45
		**LN8**	**2344**	lamellated calcareous shale	2242	20	18	46.75	3.09	0.74	1.80
		**DN12**	**2601.04**	lamellated calcareous shale	917	42	20	22.90	2.03	0.73	1.70
		**DN12**	**2600.45**	lamellated calcareous shale	2406	17	14	89.91	3.70	0.78	1.94
		**Z71**	**1676.13**	lamellated calcareous shale	706	68	26	14.54	1.75	0.53	1.42
		**DN5**	**1678.5**	lamellated calcareous shale	862	15	26	30.17	1.85	0.78	1.63
		**LN1**	**2185.9**	lamellated calcareous shale	808	25	24	9.89	0.81	0.80	1.17
		**LN1**	**2192.18**	silty shale	2462	26	18	85.01	3.05	0.70	1.79
		**Z25**	**2164.29**	lamellated calcareous shale	1337	37	25	48.98	2.32	0.70	1.63
		**Z25**	**2168.74**	lamellated calcareous shale	953	37	28	29.50	1.93	0.68	1.54
		**ZN9**	**1709.74**	lamellated calcareous shale	989	18	35	35.89	2.08	0.77	1.69
		**DN18**	**934.26**	lamellated calcareous shale	711	67	32	16.28	1.81	0.57	1.81
		**DN18**	**939.56**	lamellated calcareous shale	1044	66	27	24.47	3.04	0.53	1.54
		**average value**	1313	41	25	33.98	2.39	0.66	1.59		

### 3.2 Test Method

The testing instrument used is the AXIOSMAX XRF spectrometer produced by Panaco in The Netherlands, which uses rhodium targets as the target material and can test various constant and trace elements, such as Fe, Si, Al, P, Ca, Cu, Ba, Cr, Zr, V, and Ni. XRF spectroscopy analyzer has the characteristics of nondestructive testing, high accuracy, and fast analysis speed. XRF can analyze solid, powder, and liquid samples and has many advantages such as being suitable for the determination of major and trace elements. The error between the XRF test results and laboratory test results is less than 15%.

Based on the characteristics of the samples, 28 shale samples were selected for the testing of major and trace elements. In order to prevent sample pollution and ensure the objectivity of test results, individuals are selected to conduct pretreatment and test on samples throughout the process. The sample pretreatment process includes putting the sample into a constant temperature oven (60 °C), taking it out 24 h later, sampling and crushing it to 200 meshes, testing it at the State Key Laboratory of Marine Geology of Tongji University after subpackaging, and cooling the sample in a dry environment to room temperature after testing.

The testing process is mainly divided into three parts: weighing, melting, and testing. The three parts of testing are carried out in the State Key Laboratory of Marine Geology of Tongji University. The steps are as follows: (1) the powder sample is dried again (2 h); (2) the fluorescence agent is heated at 600 °C for 2 h, cooled it in an oven, and poured it into the original bottle, and the date is marked; (3) each test sample composed of 7.0000 g of fluorescence agent and 0.70000 g of mudstone sample is thoroughly mixed; (4) the mixed test sample is poured into a clean 50 mL Pt crucible, two drops of 20 g/50 mL lithium bromide and two drops of 30% hydrogen peroxide are added and melted it in a 1050 °C melting prototype for 8 min. After hearing a “drop” sound, the Pt plate is placed in a swinging working state and shaken for 4 min. After the shaking is completed, the instrument entering a “resting” state is awaited. After the settling is completed, the crucible is taken out, and the sample is evenly poured into a Pt plate , it is left to cool in front of the furnace (to prevent sudden cooling); (5) after cooling, the melted sheet is placed on an AXIOSMAX XRF spectrometer produced by Panaco, The Netherlands, for testing. The precision of constant (%) – trace (ppm) RSD % (major and trace elements) was 0.1–1.0.

## 4 Results

### 4.1 Lithology and Mineral Composition

**Figure 2:**
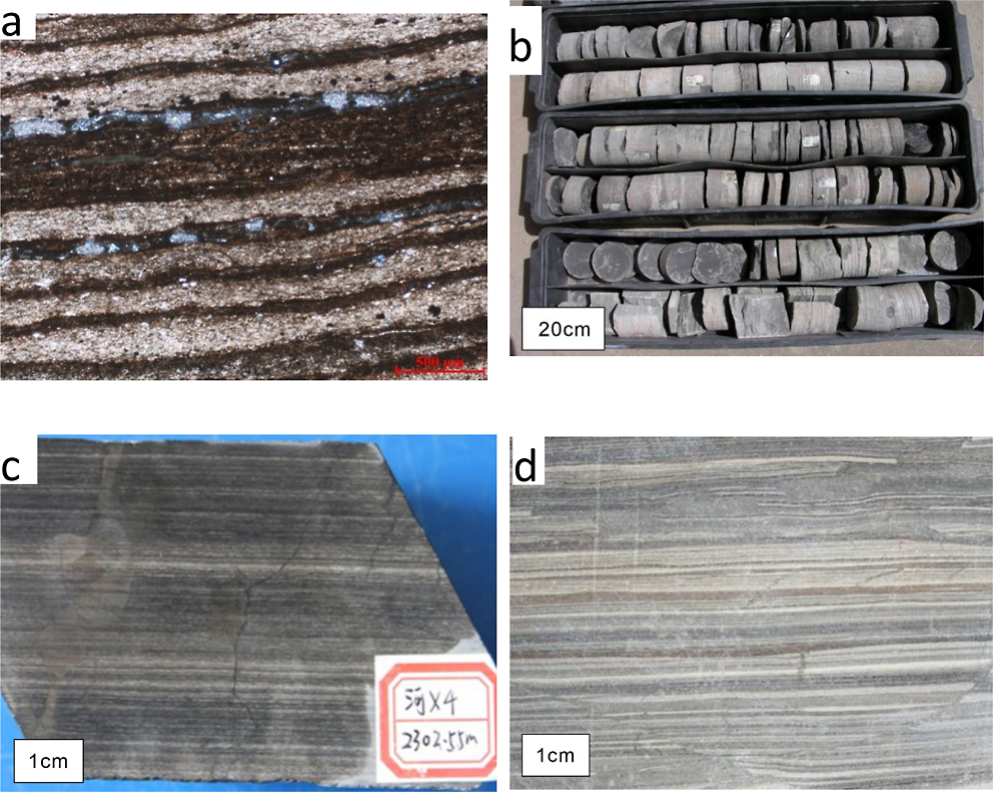
Laminated calcareous shale(E_1_f_2_) in the Subei Basin (photograph courtesy of Ms. Jianling Huang. Copyright 2023). (a)Well Zh 4, 1588.3 m, “four peaks” (E_1_f_2_), laminated calcareous shale, with shale layers enriched in asphalt and organic matter. (b) Well zh 4, 1590–1596 m, “four peaks” (E_1_f_2_) shale core. (c) Well HX4, 2302.55 m, “seven peaks” (E_1_f_2_), interbedding of grayish black lamellar calcareous shale and thin gray siltstone. (d)Well W5, 1936.62 m, “seven peaks” (E_1_f_2_), interbedding of grayish black lamellar calcareous shale and thin gray siltstone.

The shale of the second section of the Paleogene Funing Formation in the Subei Basin is mainly composed of grayish black thick shale, laminated calcareous shale, and thin-layer argillaceous siltstone in which the grayish black layer is a thin layer of organic-rich shale. The grayish black laminated calcareous shale develops horizontal bedding, and due to the changes in the content and composition of calcium in the shale, it is further divided into laminated calcareous shale and laminated dolomite shale (Figure 2).

**Figure 3:**
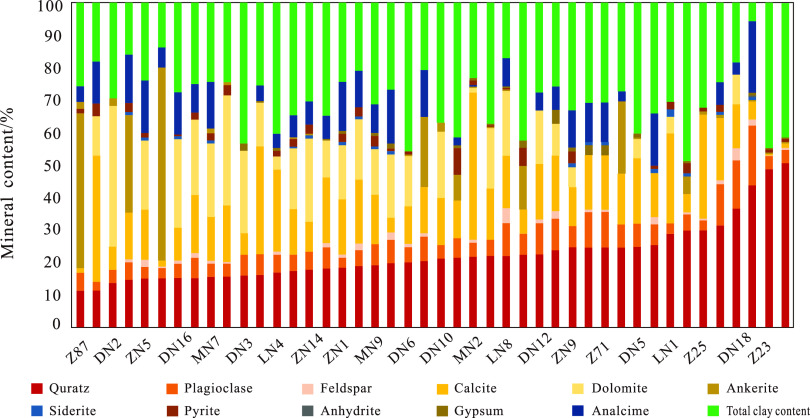
Mineral component content of laminated shales in the second section of the Funing Formation in the Gaoyou Sag.

Data analysis shows that the average content of clay minerals in the second section shale of the Funing Formation in the Gaoyou Sag is 43.3%, with felsic minerals accounting for 22.6% and carbonate minerals accounting for 34.1%. The relative content of montmorillonite in clay minerals is 12–97%, with an average of 8.5%. The relative content of illite is 4–51%, with an average of 27.6%. The relative content of kaolinite is 3–32%, with an average of 6.2%. The relative content of chlorite ranges from 2 to 37%, with an average of 11.6%. The relative content of mixed layer of illite and montmorillonite ranges from 3 to 77%, with an average of 35.3%. In felsic minerals, the quartz content ranges from 1.3 to 61.9%, with an average of 32.5%; the relative content of feldspar ranges from 36 to 48%, with an average of 14.98%; the calcite relative content of carbonate minerals is 2.6– 64.3%, with an average of 14.8%; the average dolomite relative content is 7.2%, and no iron calcite is developed. In addition, there is a certain amount of analcime and a limited amount of pyrite (Figure 3).

### 4.2 Paleoclimate of the Shale in the Second Section of the Funing Formation

In organic-rich shale, the Cu element is a life element related to life growth, and its enrichment degree usually reflects the degree of life substance eruption in the sediment.^17−19^ The ancient climate can affect the weathering intensity and rock composition of the parent rock, as reflected in the Sr/Cu element ratio of the sedimentary shale, which can better indicate the ancient climate. The Sr/Cu ratio of 1.3–5.0 indicates a warm and humid climate; the Sr/Cu ratio > 5.0 indicates a dry hot climate.

**Figure 4:**
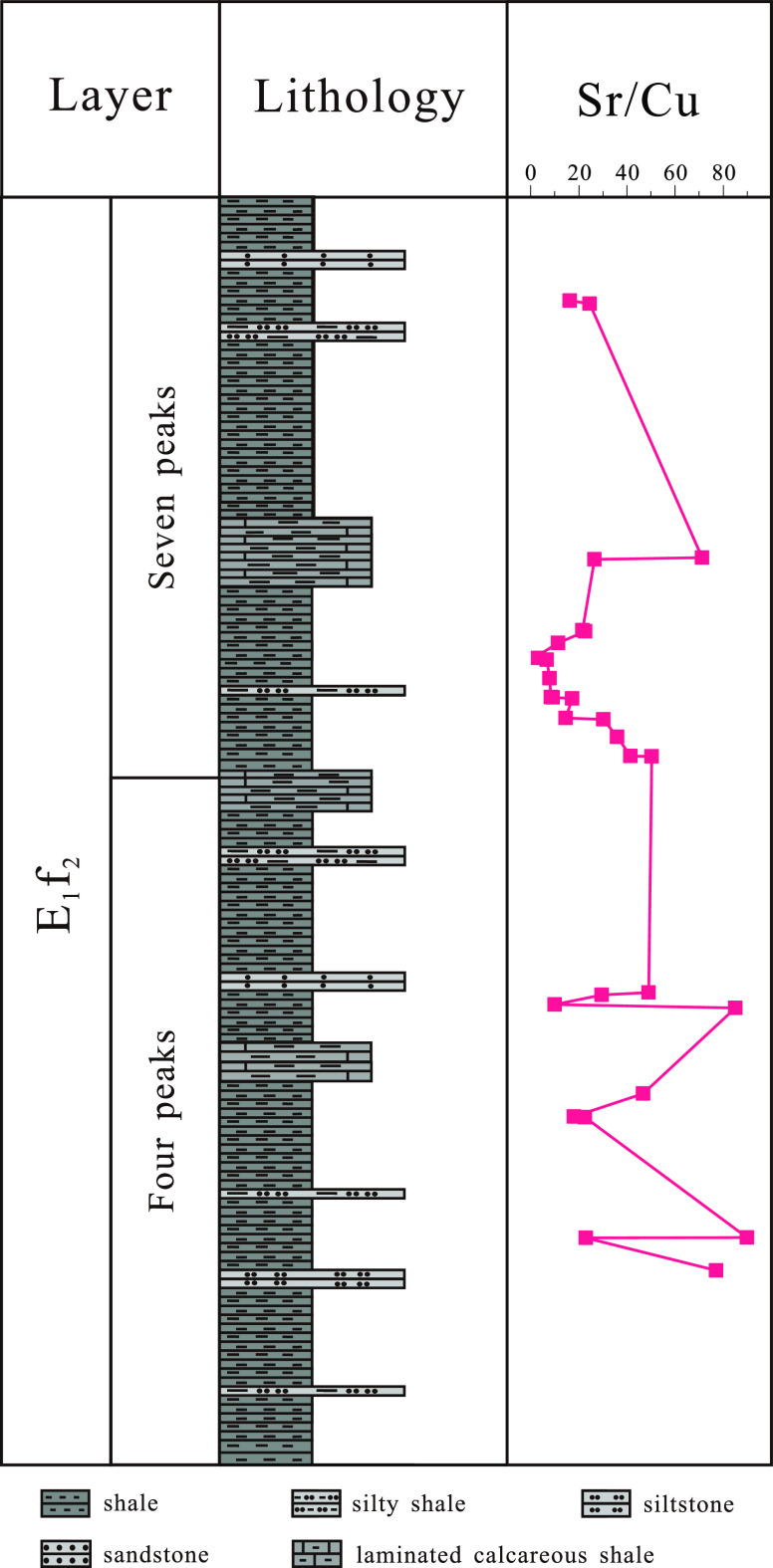
Sr/Cu ratio curve of shale in the second section of the Funing Formation in the Gaoyou Sag.

By analyzing the calculation results of the Sr/Cu ratios of 14 shale samples collected from the lower “four peaks” section of the Paleogene Funing Formation in the Gaoyou Sag, it was found that the Sr/Cu ratios ranged from 20.83 to 62.20, with an average value of 34.57. The results showed that 85.7% of the test samples had Sr/Cu ratios greater than 5 (Figure 4), reflecting the sedimentary period of the shale in the Funing Formation in the Gaoyou Sag, and the paleoclimate was mainly dry and hot.

**Figure 5:**
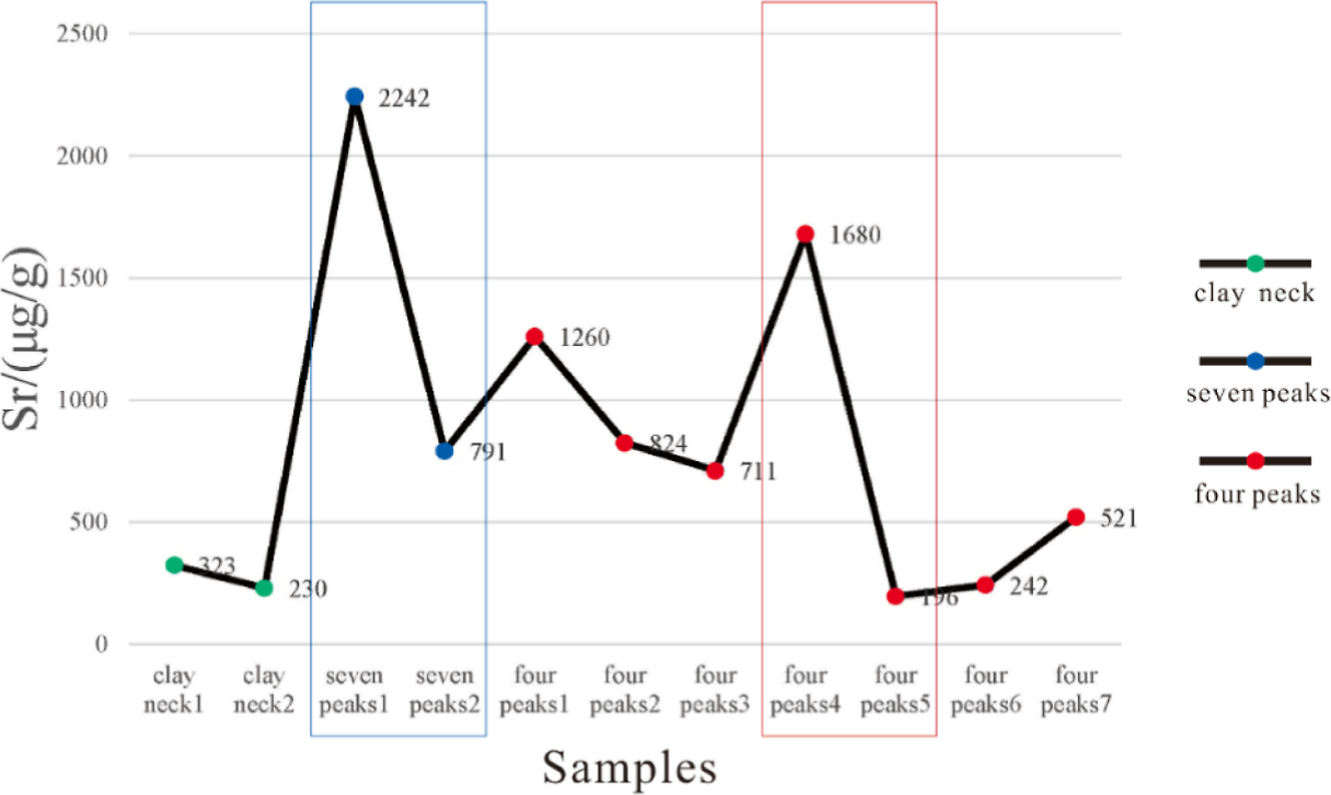
Changes in strontium (Sr) element content in the shale of the second section of the Funing Formation in the Gaoyou Sag.

Through the calculation and analysis of the Sr/Cu ratio of 15 shale samples from the middle section of the Funing Formation in the Gaoyou Sag, it was found that the Sr/Cu element ratio ranged from 9.89 to 898.91, with an average value of 33.98. The results showed that the Sr/Cu ratio of all test samples was greater than 5 (Figure 5), reflecting the sedimentary period of the “seven peaks” sublayer of shale in the middle section of the Funing Formation in the Gaoyou Sag, and the paleoclimate was dry and hot. From the perspective of Sr content in the Gaoyou Sag, the overall climate during the sedimentary period of the “four peaks” and “seven peaks” sublayer of shale in the lower and middle parts of the Funing Formation in the Gaoyou Sag is dry, with two high values of elemental Sr appearing, reflecting the high values of two dry climates. The development of underground core of laminated calcium shale also confirms that a dry environment is conducive to the formation of carbonate rock laminates.

### 4.3 Paleosalinity of Shale in the Second Section of the Funing Formation

The abundance of the Sr element can be used for qualitative judgment of water salinity. The abundance of Sr elements in saline water is generally 800–1000 ppm, in brackish water, it is generally 300–800 ppm, and in freshwater, it is 100–300 ppm.^20−22^ The strontium/barium value (Sr/Ba) can be used to restore ancient salinity based on the chemical properties of Sr and Ba, but the migration ability of Sr is higher than that of Ba, making it easier to migrate to deep ocean depths. In freshwater sediments, when Ba^2+^ meets SO_4_^2–^-rich ones, it is easy to form BaSO_4_ for precipitation. A large number of studies have shown that the Sr–Ba value in shale is an effective salinity indicator. A value greater than 1 is usually considered a saline water environment, a value less than 0.6 is a terrestrial brackish water or freshwater environment, and a value between 0.6 and 1 is a semi saline water environment.^23^ This study mainly uses trace element content and ratio to analyze the paleosalinity of the lake basin during the sedimentary period of the “four peaks” and “seven peaks” shale layers in the second section of the Funing Formation in the Subei Basin.

#### 4.3.1 Trace Element Content of Shale in the “Four Peaks” Sublayer

**Figure 6:**
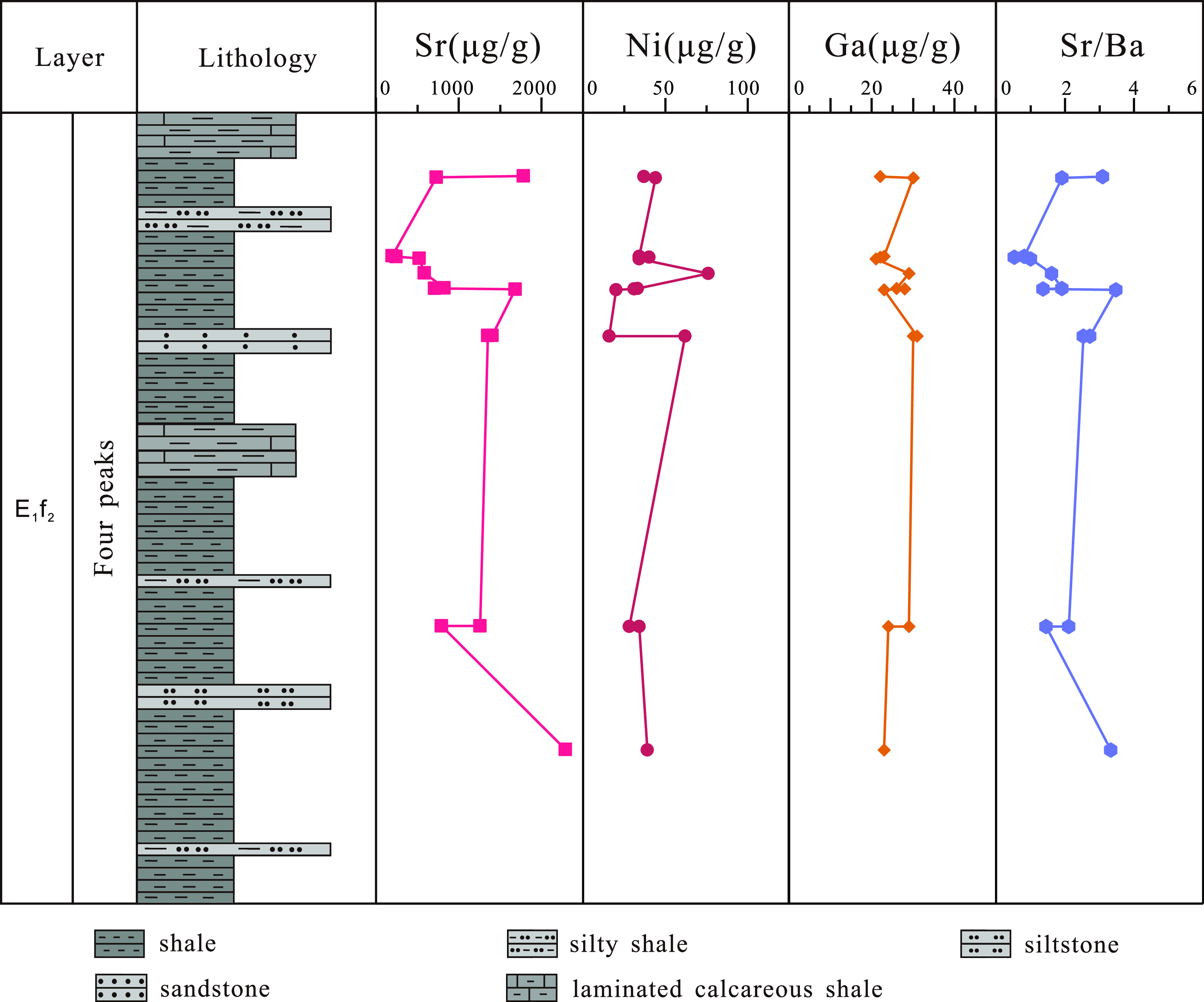
Trace element test data of the “four peaks” sublayer shale in the second section of the Funing Formation in the Gaoyou Sag.

Statistical analysis of 14 shale samples from the “four peaks” sublayer of the second section of the Funing Formation in the Paleogene of the Gaoyou Sag (Figure 6), with the elemental Sr content ranging from 196 to 2288 μg/g, with an average of 1026 μg/g, and an average value greater than 800 μ g/g. The element Ni content is 16 μg/g—76 μg/g, and the average Ni content is 38 μg/g; the content of element Ga is 21–31 μg/g, and the average Ga content is 26 μg/g, which is greater than 17 μg/g. Based on the content of Sr, Ni, and Ga elements, it is determined that the sedimentary period of the “four peaks” shale in the second section of the Funing Formation of the Paleogene in the Gaoyou Sag constituted a brackish water sedimentary environment.

Similarly, through trace element analysis of 14 shale samples, it was found that the Sr/Ba element ratio ranged from 0.54 to 3.48, with an average value of 2.00. The average value of the measured data was greater than 1. Therefore, based on the characteristics of the Sr/Ba element ratio, it can be inferred that the sedimentation period of the “four peaks” sublayer in the second section of the Funing Formation of the Paleogene in the Gaoyou Sag mainly constituted a continental brackish water environment, which is consistent with the determination of Sr, Ni, and Ga element content.

#### 4.3.2 Trace Element Content of Shale in the “Seven Peaks” Sublayer

**Figure 7:**
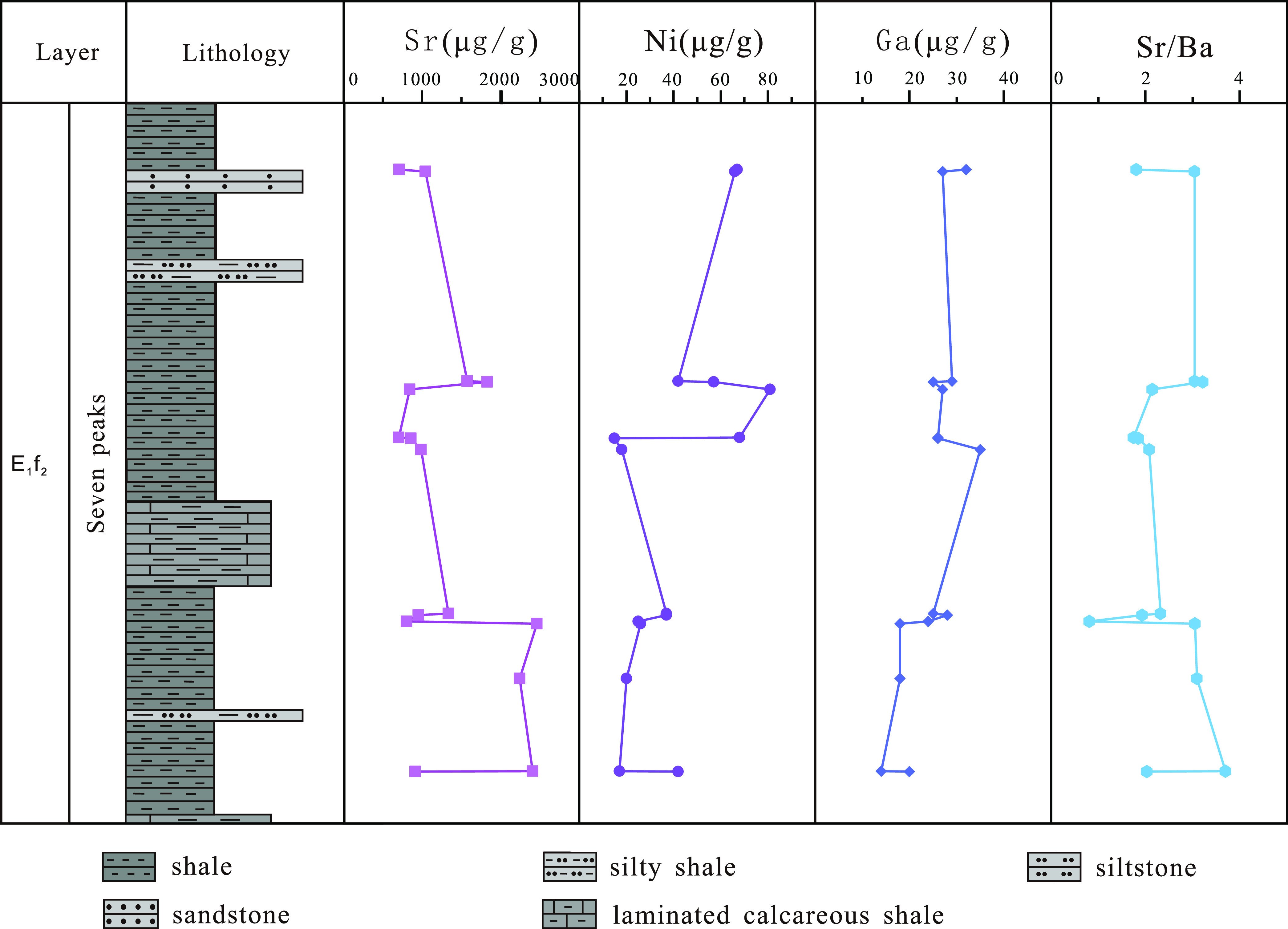
Trace element test data of the “seven peaks” sublayer shale in the second section of the Funing Formation in the Gaoyou Sag.

According to the statistics of the trace elements in 15 shale samples from the “seven peaks” sublayer of the second section of the Funing Formation in the Paleogene of the Gaoyou Sag (Figure 7), the Sr element content ranges from 706 to 2462 μg/g, with an average of 1313 μg/g; the Ni element content is 15–81 μg/g, with an average of 41 μg/g, and most samples contain greater than 20 μg/g. The content of the Ga element is 14–35 μg/g, with an average of 25 μg/g, and most samples contain greater than 17 μg/g. Based on the content of Sr, Ni, and Ga elements, it is determined that the “seven peaks” sublayer in the second section of the Funing Formation in the Gaoyou Sag belongs to the brackish water sedimentary environment.

Similarly, trace element analysis of 15 shale samples from the “seven peaks” sublayer of the Funing Formation in the Gaoyou Sag showed that the Sr/Ba element ratio ranged from 0.81 to 3.70, with an average value of 2.39. The average value is greater than 1, indicating that the sedimentary period of the “seven peaks“ sublayer was mainly in a continental brackish water environment.

### 4.4 Oxidation–Reduction Conditions during the Sedimentation of Shale in the Second Section of the Funing Formation

The ratio of trace elements can effectively indicate the oxidation reduction conditions of sedimentary environments.^24^ By using V/(V + Ni) and V/Cr methods, the oxidation–reduction conditions of the “four peaks” and “seven peaks” sublayers of shale in the second section of the Funing Formation of the Paleogene in the Gaoyou Sag were determined during the sedimentary period.

Trace elements such as vanadium, nickel, and chromium are mainly adsorbed and precipitated by colloidal particles or clay in shale. V is easily adsorbed under reducing conditions, while Ni and Cr are easily enriched under reducing conditions. Therefore, the V/(V + Ni) and V/Cr ratios of elements can indicate the redox environment of sedimentary water bodies.^25,26^ Previous studies have shown that in anaerobic reduction environments(Table 2), the V/(V + Ni) ratio is greater than 0.84, ranging from 0.84 to 0.89, and the V/Cr ratio is greater than 4.25. In anaerobic and subreducing environments with weak water stratification, the V/(V + Ni) ratio ranges from 0.60 to 0.84, and the V/Cr ratio ranges from 2.00 to 4.25. In an oxidizing environment, the V/(V + Ni) ratio is less than 0.60, and the V/Cr ratio is less than 2.00.^27−30^

**Table 2:** Classification of Oxidation–Reduction Sedimentary Environment Standards by the Ratio of Trace Elements^25,26^

	element ratio	
redox environment	V/(V + Ni)	V/Cr
anaerobic reducing environment	>0.84	>4.25
anaerobic subreducing environment	0.60–0.84	2.00–4.25
oxidizing environment	<0.60	<2.00

**Figure 8:**
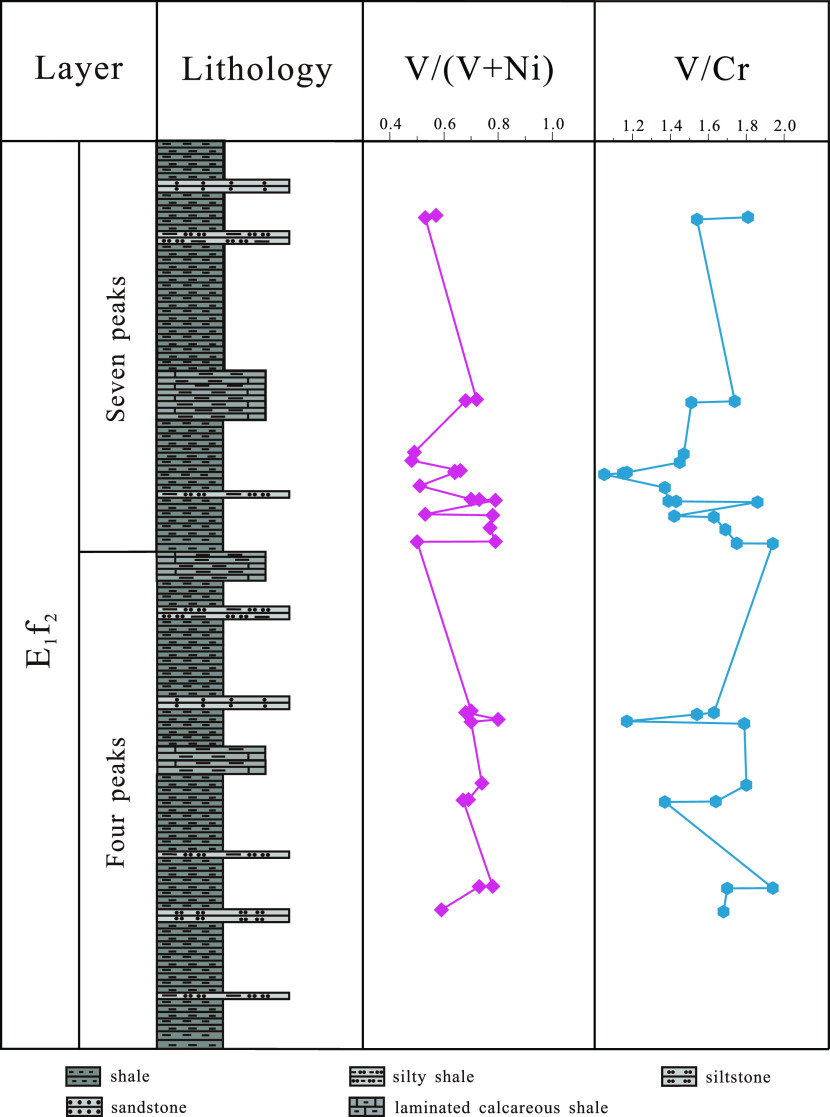
Analysis results of oxidation–reduction conditions for trace element testing in the shales of Paleogene in the Gaoyou Sag.

The trace element analysis results of 15 shale samples collected from the “four peaks” sublayer of the Funing Formation in the Paleogene of the Gaoyou Sag (Figure 8) show that the V/(V + Ni) ratio is 0.51–0.79, with an average value of 0.66 and all ratios being less than 0.84. The V/Cr ratio is 1.05–1.94, with an average value of 1.51, and all ratios are less than 2. According to the discriminant indicators, it is indicated that the sedimentary water of the “four peaks” shale in the second section of the Funing Formation in the Gaoyou Sag is a weakly stratified oxidation–weak reduction transitional environment.

### 4.5 Paleowater Depth during the Sedimentation of Shale in the Second Section of the Funing Formation of the Paleogene in the Gaoyou Sag

The restoration of ancient water depth can be comprehensively judged by lithology, sedimentary structure, and paleontological data. At the same time, modern sediment-element geochemical research shows that due to the differentiation of elements in sedimentation, the accumulation and dispersion of elements have a certain relationship with the offshore distance.^31−33^ This property can also be used to judge the ancient water depth.

It is generally believed that shallow lake–semi deep lake is the dominant facies belt of laminated calcareous shale and fine siltstone deposition, and the theoretical water depth is 20–100 m.^34−37^ The laminated calcareous shale of the second section of the Paleogene Funing Formation in the Gaoyou Sag is mainly black, dark gray, and grayish white and rich in organic matter. The laminated calcareous shale is mixed with a few siltstone laminae, and the horizontal bedding and foliation are developed, belonging to typical semi deep lake deposits.

From the TOC content of laminated calcareous shale in the Gaoyou Sag, the average TOC of the “seven peaks” shale in the second section of the Funing Formation of the Paleogene is 1.97%, which is greater than the 1.37% TOC content of the “four peaks” shale. This reflects that the lake water was shallow when the “four peaks” shale was deposited, while the lake water was deep when the “seven peaks” shale was deposited.

### 4.6 Paleoenvironmental Results

**Figure 9:**
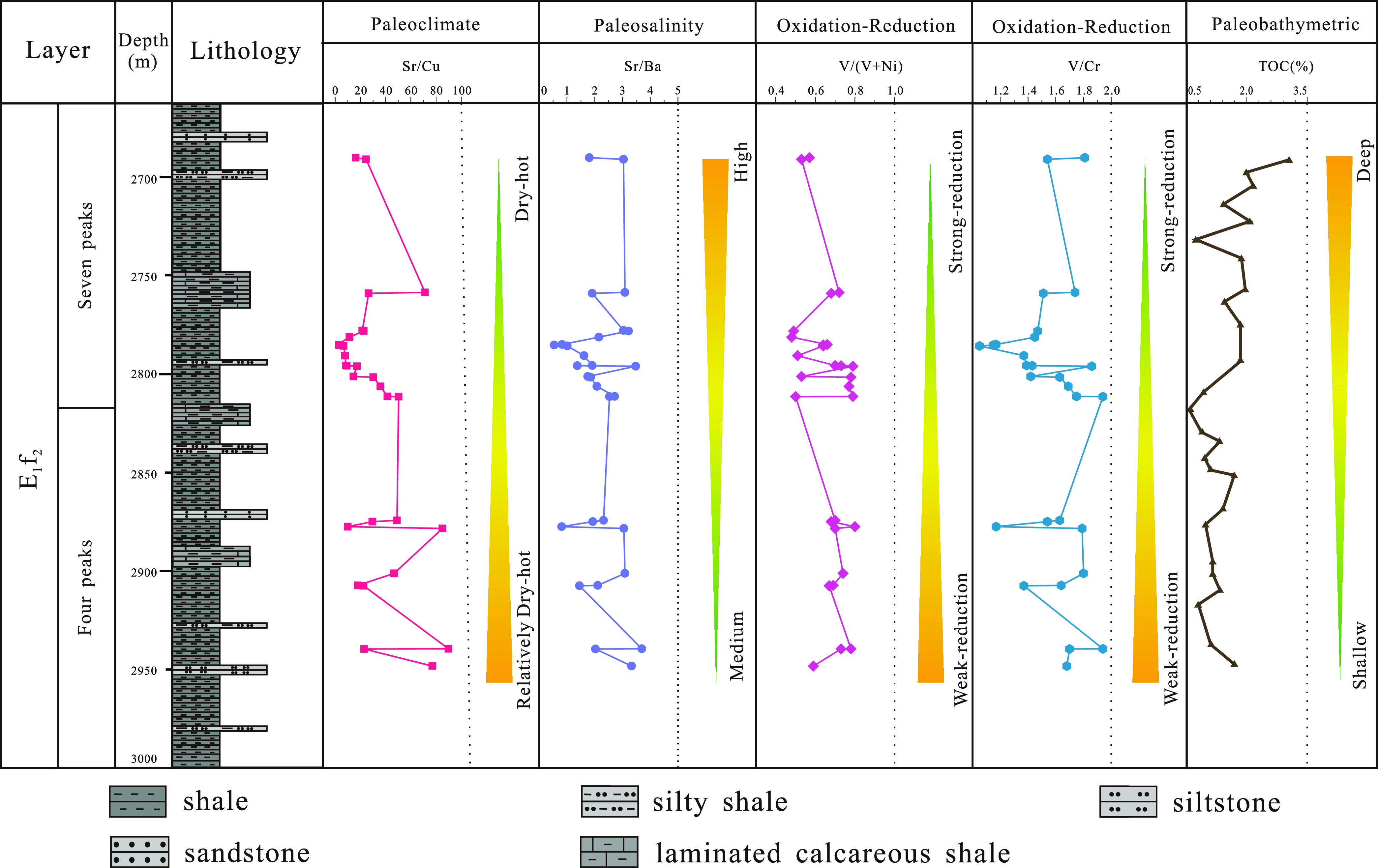
Evolution of the paleoenvironment of the “four peaks” and “seven peaks” shales in the second section of the Funing Formation in the Gaoyou Sag.

The sedimentary period of the “four peaks” and “seven peaks” sublayers in the second section of the Funing Formation of the Paleogene in the Gaoyou Sag was characterized by dry and hot lake sedimentation in the Subei Basin. The evolution of the lake paleoenvironment during the sedimentary period of the “four peaks” and “seven peaks” sublayers can be summarized from a time scale (Figure 9). It can be concluded that during the sedimentation period of the “four peaks” and “seven peaks” sublayers of shale, seawater intrusion occurred in the lake, and the lake surface rose, resulting in a relatively stable sedimentary environment. The ancient salinity of the lake water gradually increases from bottom to top, and the ancient salinity of the lake gradually transitions from semi saline water during the sedimentation of the “four peaks” sublayer to saline water during the sedimentation of the “seven peaks” sublayer. The entire sedimentary period of the second section of the Funing Formation is mainly characterized by a weak reduction–strong reduction environment. During the sedimentary period of the “four peaks” sublayer shale, the lake water depth is shallow, which is a weak reduction environment. The sedimentary organic matter in the shale is less preserved, while during the sedimentary period of the “seven peaks” sublayer shale, the lake water depth is deep, which is a strong reduction environment. The evolution of ancient climate shows significant climate changes during the deposition of the sublayer from the “four peaks” to the “seven peaks”, and the dry hot climate gradually strengthens. The ancient water depth gradually deepened from the “four peaks” to the “seven peaks” sedimentary period. There were rare to no biological remains in the sedimentary rock, and there was no obvious hydrodynamic sedimentary structure. The main features were horizontal bedding and massive bedding, reflecting the sedimentary process of shale from the “four peaks” to the “seven peaks”. The impact of the transgression was further strengthened, and the lake level continued to rise.

## 5 Discussion

Based on the above research results, we restored the paleosedimentary environment and its evolution through the analysis of shale constants and trace elements deposited in paleolakes. However, there is further research and discussion on the formation and changes of shale laminae with the following problem values.

(1) What factors are most related to the original origin of the laminae and the paleolake environment? The laminated calcareous shale in the shale of the second section of the Funing Formation of Paleogene in the Subei Basin has a thickness of 0.01–0.1 mm which is also the smallest or thinnest original sedimentary layer in the core. According to the analysis of ordinary and trace elements in the shale, it has been concluded that the shale of the second section of the Funing Formation in the Subei Basin was formed by frequent lake level fluctuations at the bottom of the lake under dry and hot climate and deep-water conditions of saline lakes, but which factor is the main one is unclear.
(2) Further research is needed on the genesis of calcareous laminae in shale. Some scholars believe that the increase of lake surface temperature will also reduce the pressure of carbon dioxide in the lake water and eventually cause the supersaturation precipitation of calcium carbonate in the water to form carbonate rock laminae. It is obvious that the calcareous laminae is a product of chemistry or biochemistry. However, in the shale of the second section of the Funing Formation of the Paleogene in the Subei Basin, the occurrence of calcareous laminae is extremely frequent, and both calcareous and dolomite laminae appear in the shale, indicating significant differences in the formation environment of the laminae.
(3) What is the relationship between the hydrodynamic forces and the formation of calcareous shale laminae? Although it is generally believed that shale is a product of sedimentation in quiet and deep-water environments, the formation of shale or calcareous laminae is clearly influenced by different environments. Further exploration and research on the role and value of hydrodynamic forces in this process are needed.

## 6 Conclusions

Based on the research on the characteristics of trace elements and their vertical changes in the second section of the Funing Formation in the Gaoyou Sag, combined with sedimentary lithologic indicators, the environmental development characteristics of paleolakes such as paleoclimate, paleosalinity, redox, and paleowater depth during shale deposition were analyzed vertically, and the following conclusions were obtained:

(1) The sedimentary environment of the second section of the Funing Formation shale in the Gaoyou sag during the sedimentation period was a dry and hot climate environment; the salinity of the lake water was that of brackish water and was relatively stable.
(2) During the sedimentary period of the second section of the Funing Formation, shale was deposited in a strong reducing environment, which is conducive to the preservation of sedimentary organic matter in the shale.
(3) The frequent changes in climate and salinity of water due to alternating dryness and hotness have led to the development of the “four peaks” sublayer at the bottom of the second section of the Funing Formation and the “seven peaks” sublayered laminated calcareous shale at the top.
(4) The deep-water reduction environment and changes in lake salinity of saline paleolakes are the result of biological breeding, sedimentation, and preservation, evolving into abundant commercially exploitable shale oil resources in laminated calcareous shale.
